# Hepatocellular carcinoma occurs frequently and early after treatment in HCV genotype 3 infected persons treated with DAA regimens

**DOI:** 10.1186/s12876-020-01249-4

**Published:** 2020-04-06

**Authors:** Ghias Un Nabi Tayyab, Shafqat Rasool, Bilal Nasir, Ghazala Rubi, Abdul-Badi Abou-Samra, Adeel A. Butt

**Affiliations:** 1 Post Graduate Medical Institute, Ameer Ud Din Medical College, Lahore, Pakistan; 2 Lahore General Hospital, Lahore, Pakistan; 3 Weill Cornell Medical College, New York, NY USA; 4 Weill Cornell Medical College, Doha, Qatar; 5 Hamad Medical Corporation, Doha, Qatar

**Keywords:** Hepatocellular carcinoma, HCV genotype 3, DAA, Pakistan

## Abstract

**Background:**

There are conflicting data regarding the risk of hepatocellular carcinoma (HCC) after direct-acting antiviral agent (DAA) treatment. Risk of HCC in HCV genotype-3 infected persons after DAA therapy is not well known.

**Methods:**

We prospectively studied HCV infected persons initiated on a DAA regimen between October 2014 and March 2017 at two centers in Pakistan. All persons were free of HCC at study initiation. HCC was confirmed based on characteristic CT scan findings. Patients were followed for 12 months after the completion of therapy.

**Results:**

A total of 662 persons initiated treatment. Median age (IQR) was 50 (41, 57) years and 48.8% were male. At baseline, 49.4% were cirrhotic, 91% were genotype 3 and 91.9% attained SVR. Treatment regimens used were: Sofosbuvir (SOF)/ribavirin (RBV)/pegylated interferon (PEG-IFN), 25.2%; SOF/RBV, 62.4%; SOF/RBV/daclatasavir (DCV), 10.6%; SOF/DCV, 2.0%. Incident HCC was detected in 42 patients (12.8%) in the 12-month period after treatment completion and was exclusively observed in those with cirrhosis. In multivariable Cox regression analysis, SVR was associated with a reduction in HCC risk (HR, 95% CI: 0.35, 0.14,0.85). In Kaplan-Meier plots by treatment regimen, those treated with SOF/RBV, SOF/RBV/DCV, or SOF/DCV regimens had a shorter HCC-free survival compared with those treated with a SOF/RBV/PEG-IFN regimen.

**Conclusion:**

In a predominantly genotype 3 cohort, incident HCC occurred frequently and early after treatment completion, and exclusively in those with pre-treatment cirrhosis. SVR reduced the risk of HCC. Treating HCV infected persons before development of cirrhosis may reduce risk of HCC.

## Background

Treatment of hepatitis C virus (HCV) has been revolutionized by the use of direct acting antiviral agents (DAAs) against HCV. These regimens are more efficacious compared with the older interferon based regimens, with sustained virologic response (SVR) rates consistently exceeding 90–95% [1–5]. Their general safety profile and tolerability is well established in randomized clinical trials and large real-world studies [6–8]. Other clear benefits of DAA-based regimens include reduction in overall mortality, reduced risk of non-liver cancers, and reduction in incidence of several extrahepatic complications including diabetes, stroke and cardiovascular disease events [9–13]. Some earlier reports suggested an association between the newer DAA-based regimens and reactivation of hepatitis B virus (HBV) infection, progression of chronic kidney disease, and early hepatic decompensation events [14, 15]. However, larger studies in a variety of settings, and with appropriate control groups have not corroborated these associations [7, 8, 16–19]. The association of DAA-regimens with hepatocellular carcinoma (HCC) is less clear. Multiple reports have suggested an increased incidence of HCC in persons treated with DAA-based regimens [20–22]. However, most of this risk was attributed to recurrence of previously treated HCC. Several other studies have found no association between DAA-based regimens and risk of incident HCC [17, 23–25]. Pakistan has one of highest rates of HCV infection, mostly associated with unsafe injection practices [26, 27]. The predominant genotype In Pakistan is genotype 3 infection, accounting for 66–72% of the infected persons [28, 29]. Newer DAA-based regimens are now widely available in Pakistan, and > 90% persons with HCV genotype 3 infection treated with these regimens attain SVR [30]. The risk of HCC among persons with HCV genotype 3 infection who are treated with newer DAA-based regimens is unknown. We undertook this to determine the association between DAA-based regimen and incident HCC in a predominantly HCV genotype 3 population in Pakistan.

## Methods

### Study setting and participants

We prospectively enrolled adult persons with HCV infection who were initiated on a DAA-based regimen between October 2014 and March 2017 at two centers in urban Pakistan. All consecutive persons who provided informed consent, were eligible for the study and free of HCC were enrolled. At baseline, a detailed history, physical examination and laboratory evaluation was performed. Liver imaging (high quality ultrasound) and serum alfa fetoprotein (AFP) levels were performed to rule out HCC at baseline. Treatment for HCV was initiated based on preferred regimens listed in the national guidelines and regimen availability at the treating center [31]. All enrolled persons were followed at monthly intervals during the course of treatment and for 24 weeks after completion of treatment, and at three-monthly intervals thereafter for at least 12 months after treatment completion. Clinical assessment, AFP levels and liver ultrasound were performed at the three-month visits. Any new hepatic decompensation event, new liver nodule on ultrasound, or an AFP increase of > 10 IU/mL were followed up by a dynamic computerized tomographic (CT) scan of the liver to rule out HCC.

### Definitions

Our primary endpoint was incident HCC, which was diagnosed on the basis of characteristic findings on CT scan of the liver during the 12 months follow up after completion of treatment. Sustained virologic response was defined as undetectable HCV RNA 12 or more weeks after completion of treatment. Presence of liver cirrhosis was determined using characteristic liver imaging findings (ultrasound or CT scan) and transient elastography using a cutoff of 12.5 kPa. Comorbidities were diagnosed by participant self-report.

### Statistical analyses

Baseline clinical characteristics were compared for persons with and without incident HCC. Mean (+ standard deviation) or median (interquartile range) were calculated for normally and non-normally distributed continuous data respectively. Multivariable Cox proportional hazards analysis was used to determine the predictors of incident HCC. Assumptions of proportionality were ascertained using Schoenfeld residuals. Kaplan-Meier curves were generated to demonstrate time to diagnosis of HCC by attainment of SVR, and HCC-free survival by treatment regimen. Log-rank test was used to determine statistical significance of the difference, with *p*-value of < 0.05 considered as a statistically significant difference.

### Ethical considerations

Institutional approvals were obtained at each site prior to study initiation. All participants provided informed consent prior to entry into the study.

## Results

A total of 662 participants were included in the study. The median age was 50 years (inter-quartile range 41,57) and 48.8% were male. **(**Table 1**)** Mean weight of the participants was 71.8 kg (standard deviation 13.1), 6.9% had hypertension, 28.2% had diabetes and 1.4% were coinfected with hepatitis B virus. Overall, 90.8% had HCV genotype 3 infection and 49.4% had cirrhosis (89.3% compensated cirrhosis; 10.7% decompensated cirrhosis). All treatment regimens included sofosbuvir (SOF) plus at least one other agent (ribavirin [RBV], pegylated interferon [PEG-IFN], daclatasvir [DCV]; Table 1). Sustained virologic response was attained in 91.9% of participants. A total of 42 cases of HCC were observed in the 12-month post-treatment period, with a median time to first diagnosis of 28 weeks (IQR 21,39). Baseline characteristics of those with and without HCC are presented in Table 2**.** Of note, all persons with incident HCC had prevalent liver cirrhosis.

**Table 1:** Baseline characteristics. *N* = 662

Variable	Result
Median age in years, IQR	50 (41,57)
Sex, % male	48.79
Weight, mean (SD) kg	71.8 (13.1)
Obesity (% obese)	16.62
Hypertension, %	6.95
Diabetes, %	28.25
Hepatitis B virus coinfection	1.36
Cirrhosis, overall %	49.40
Cirrhosis compensated, %	89.27
Cirrhosis decompensated, %	10.73
HCV RNA, median log_10_ IU/mL (IQR)	5.90 (5.20,6.45)
HCV genotype	
HCV genotype 3	90.79
HCV genotype non-3	9.21
Sustained virologic response, %	91.90
Incident HCC, %	6.34
Median time to HCC, weeks (IQR)	28 (21,39)
Treatment regimen, %	
SOF/RBV/PEG-IFN	25.23
SOF/RBV	62.24
SOF/DCV/RBV	10.57
SOF/DCV	1.96

*IQR* inter-quartile range, *HCC* hepatocellular carcinoma, *SOF* sofosbuvir, *RBV* ribavirin, *PEG-IFN* pegylated interferon, *DCV* daclatasvir;

**Table 2:** Baseline characteristics of persons with and without incident HCC

Variable	With HCC (*N* = 42)	Without HCC	*P*-value
Mean age, (SD)	57.4 (8.4)	48.6 (11.3)	< 0.0001
Median age, IQR	58 (53,65)	50 (40,56)	< 0.0001
Sex, % male	45.24%	49.03%	0.63
Weight, mean (SD) kg	67.4 (13)	72.1 (13)	0.02
Obesity (% obese)	11.90%	16.93%	0.40
Hypertension, %	7.14%	6.93%	0.96
Diabetes, %	47.60%	26.90%	0.004
Hepatitis B virus coinfection	0.30%	1.06%	0.09
Cirrhosis, %	100.00%	45.97%	< 0.0001
Cirrhosis decompensated, %	30.95%	9.35%	< 0.0001
HCV RNA, median log_10_ IU/mL (IQR)	6.30 (5.84,6.51)	5.87 (5.20,6.45)	0.24
HCV genotype 3	95.2	90.5	0.41
Sustained virologic response, %	87.5	92.2	0.29
Median time to HCC, weeks (IQR)	28 (21,39)	N/A	–
Treatment regimen, %			0.0006
SOF/RBV/PEG-IFN	2.4	26.8	
SOF/RBV	71.4	61.6	
SOF/DCV/RBV	23.8	9.7	
SOF/DCV	2.4	1.9	

*IQR* inter-quartile range, *HCC* hepatocellular carcinoma, *SOF* sofosbuvir, *RBV* ribavirin, *PEG-IFN* pegylated interferon, *DCV* daclatasvir;

Increasing age was associated with a higher risk of incident HCC (HR 1.75; 95% CI 1.29,2.37) in Cox proportional hazards analysis. **(**Table 3**)** While the hazards ratios for all DAA-based regimens demonstrated a higher risk compared with SOF/PEG-IFN/RBV regimen, only SOF/DCV/RBV regimen was statistically significantly associated with a higher risk of incident HCC. However, the 95% confidence intervals for all hazards ratios were very wide, making an assumption of true association relatively difficult. Sustained virologic response was associated with a significant reduction in the risk of incident HCC (HR 0.35, 95% CI 0.14,0.85).

**Table 3:** Predictors of development of hepatocellular carcinoma (multivariable Cox regression model)

Variable	HR (95% CI)	*P*-value
Age, per 10 year increase	1.75 (1.29,2.37)	0.0003
Male sex	1.21 (0.63,2.35)	0.57
Weight, each 5 kg increase	0.83 (0.73,0.96)	0.01
Hypertension	1.01 (0.3,3.42)	0.99
Diabetes	1.46 (0.76,2.79)	0.26
HCV genotype 3 (comparator genotype non-3)	1.42 (0.34,5.99)	0.64
Sustained virologic response	0.35 (0.14,0.85)	0.02
Treatment regimen		
SOF/RBV/PEG-IFN (comparator)		
SOF/RBV	6.75 (0.89,51.1)	0.06
SOF/DCV/RBV	17.32 (2.14,140.36)	0.01
SOF/DCV	8.63 (0.53,141.25)	0.13

*SOF* sofosbuvir, *RBV* ribavirin, *PEG-IFN* pegylated interferon, *DCV* daclatasvir

**Fig. 1:**
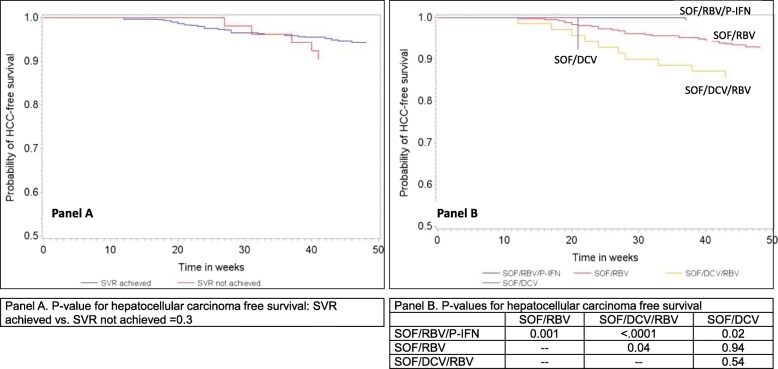
Kaplan-Meier curves showing time to hepatocellular carcinoma by attainment of SVR (**a**), and hepatocellular carcinoma free survival by treatment regimen (**b**)

In Kaplan-Meier analysis, time to first HCC diagnosis was not different among those who attained SVR vs. those who did not. **(**Fig. 1a; log-rank *p*-value = 0.3) Hepatocellular carcinoma-free survival was significantly different between various DAA-based treatment regimens. (Fig. 1b The combination regimen including PEG-IFN and RBV had significantly longer HCC-free survival compared with other DAA-based regimens without PEG-IFN/RBV.

### Additional analyses

There were 9 persons with HBV coinfection. We repeated all analyses after removing those 9 persons with HBV coinfection. Baseline characteristics of the updated cohort (*N* = 653) are presented in **supplementary Table** **1**. After removing the 9 persons with HBV coinfection, 40 persons had a diagnosis of HCC. Baseline characteristics of these 40 persons with HCC and 613 persons without HCC are presented in **supplementary Table** **2**. Finally, the predictors of development of HCC in the updated cohort are presented in **supplementary Table** **3**. All results in the updated cohort were similar to the main results.

## Discussion

In this large study of persons with predominantly HCV genotype 3 infection, incident HCC was observed frequently and early after completion of treatment with a DAA-based regimen.

The association between DAA-based regimens and development of HCC is complicated and poorly understood. Early uncontrolled studies suggested a higher risk of HCC in persons treated with DAA regimens, which was more pronounced for recurrence of HCC after previous successful treatment rather than de novo development of HCC. More recent larger studies with improved design and appropriate control groups have failed to substantiate this association. In fact, DAA-based regimens have been associated with a similar or reduced risk of incident or de novo HCC when compared with untreated persons or those treated with older interferon-based regimens [17, 23, 32]. There are scant and conflicting data regarding the risk of de novo HCC in persons with HCV genotype 3 infection who are treated with DAA-based regimens. While at least one study has reported a lower incidence of HCC in persons with HCV non-genotype 1 infection, another large national study observed a clear association between HCV genotype 3 infection and HCC cirrhosis [33, 34]. Neither studies addressed the role of DAA-based regimens upon the risk of HCC. In our analysis, HCV genotype 3 infected persons treated with a DAA-based regimen were at a higher numerical risk of incident HCC, though this association did not reach statistical significance. This is perhaps due to the small number of persons with non-genotype 3 infection. Future studies with larger comparison group with non-genotype 3 infection may help clarify this association.

All cases of HCC were observed in those with prevalent cirrhosis. This is consistent with some previous studies, though other studies have clearly demonstrated that HCC can occur in the absence of pre-existing cirrhosis [35]. Regardless, persons with cirrhosis constitute the highest risk group for development of HCC and require regular and frequent screening for HCC. Additionally, an association between metabolic factors, particularly diabetes, has been reported in many studies [36]. Curiously, a protective effect has been demonstrated with higher weight. Our study shows a higher risk of HCC in persons with diabetes, though this did not reach statistical significance, and a lower risk of HCC with increasing weight. Apparent protective effect of increasing weight may be the result of a “healthy person” phenomenon where persons with normal or increased weight are less likely to have chronic underlying diseases; conversely, weight loss in persons with HCC may have unmasked this spurious association.

The benefit of attaining SVR is clearly described in literature. Clearance of the virus from circulation, as evidenced by undetectable HCV RNA, is associated with reduced mortality and a reduced risk of several complications [9, 10, 12, 13, 32, 37–39]. We found the attainment of SVR to be strongest protective factor against development of HCC. This underscores the need for increasing access to early and effective treatment, with multiple anticipated benefits.

The role of specific regimen(s) upon the risk of incident HCC is less clear. While we observed a higher risk among persons treated with SOF/DCV/RBV compared with SOF/PEG-IFN/RBV regimen, the 95% confidence interval was very wide (HR 17.32; 95% CI 2.14,140.36) to make any clinical practice change recommendations. The risk with other regimens (SOF/RBV; SOF/DCV/RBV; SOF/DCV) was also high, but these associations did not reach statistical significance. Possible reasons for a lower observed risk in persons treated with an interferon-containing regimen could be lesser fibrosis in these persons, inherent immune modulating property of interferon, or other differences in baseline characteristics of patients. Whether inclusion of PEG-IFN reduces the risk of HCC in persons with HCV genotype 3 infection warrants further study.

Screening for HCC is critical in persons with HCV and cirrhosis, though some persons may develop HCC in the absence of cirrhosis. Alfa fetoproteion levels and liver imaging are often used to screen patients for HCC. While computed tomography or magnetic resonance imaging are more sensitive and specific for HCC, ultrasound is cheaper and more readily available, particularly in resource limited settings. The sensitivity of ultrasound varies from 84 to 94% for all stage HCC and 47–63% for early stage HCC [40, 41]. Addition of AFP has been shown to increase sensitivity in some, but noth other studies. It is potentially possible that some of the early detected tumors in our study subjects were actually present even before treatment started and were not recognized by our screening techniques.

The strengths of our study include its prospective design and large number of participants. Limitations include a small non-genotype 3 comparison group, lack of validated measures of certain other risk factors for HCC, e.g. non-alcoholic fatty liver disease, aflatoxin exposure. It must also be noted that the treatment regimens used during the study time period do not conform with the most recent treatment guidelines, though the overall efficacy and benefits of treatment are still valid.

## Conclusions

In conclusion, persons with HCV genotype 3 infection treated with DAA-based regimens experience frequent and early occurrence of de novo HCC after completion of treatment. The risk is exclusively observed in those with existing liver cirrhosis. Attainment of SVR significantly reduces that risk. The role of specific DAA-based regimens upon this risk is less clear and warrants further study. Persons with HCV genotype 3 infection treated with DAA-based regimens, particularly those with cirrhosis, should be screened for HCC early and frequently after completion of treatment.

## Supplementary information

**Additional file 1 Supplementary Table 1.** Baseline characteristics excluding 9 persons with hepatitis B virus coinfection. **Supplementary Table 2.** Baseline characteristics of persons with and without incident HCC excluding 9 persons with hepatitis B virus coinfection. **Supplementary Table 3.** Predictors of development of HCC (multivariable Cox regression model) excluding 9 persons with hepatitis B virus coinfection.

## Data Availability

Subject level data are not available to the public. Aggregate data may be shared if all regulatory and legal approvals have been obtained by the requesting entity/person(s). Contact the corresponding author (Adeel A. Butt) with any request along with approvals.

## Abbreviations

AFP: Alfa fetoprotein
CT: Computerized tomographic scan
DAA: Direct acting antiviral agents
DCV: Daclatasvir
HBV: Hepatitis B virus
HCC: Hepatocellular carcinoma
HCV: Hepatitis C virus
PEG-IFN: Pegylated interferon
RBV: Ribavirin
SOF: Sofosbuvir
SVR: Sustained virologic response
