# Supplementary material for: Hepatocellular carcinoma occurs frequently and early after treatment in HCV genotype 3 infected persons treated with DAA regimens
Source: BMC Gastroenterol. 2020 Apr 6;20:93. doi: 10.1186/s12876-020-01249-4 (PMC7137260; doi:10.1186/s12876-020-01249-4)
Supplement: Supplementary file 1 — Additional file 1 Supplementary Table 1. Baseline characteristics excluding 9 persons with hepatitis B virus coinfection. Supplementary Table 2. Baseline characteristics of persons with and without incident HCC excluding 9 persons with hepatitis B virus coinfection. Supplementary Table 3. Predictors of development of HCC (multivariable Cox regression model) excluding 9 persons with hepatitis B virus coinfection. [file 12876_2020_1249_MOESM1_ESM.docx]

Supplementary table 1. Baseline characteristics excluding 9 persons with hepatitis B virus coinfection.

| Variable | Result  N=653 |
| --- | --- |
| Median age, IQR | 50.0 (41,56) |
| Sex, % male | 48.85 |
| Weight, mean (SD) kg | 71.8 (13.0) |
| Obesity (% obese) | 16.69 |
| Hypertension, % | 7.04 |
| Diabetes, % | 28.33 |
| Hepatitis B virus coinfection |  |
| Cirrhosis, % | 49.31 |
| Cirrhosis compensated, % | 89.43 |
| Cirrhosis decompensated, % | 10.57 |
| HCV RNA, median log_10_ IU/mL (IQR) | 5.9 (5.2,6.5) |
| HCV genotype |  |
| HCV genotype 3 | 90.81 |
| HCV genotype non-3 | 9.19 |
| Sustained virologic response, % | 91.78 |
| Incident hepatocellular carcinoma, % | 6.13 |
| Median time to HCC, weeks (IQR) | 27.5 (20.5,38.5) |
| Treatment regimen, % |  |
| SOF/RBV/PEG-IFN | 25.11 |
| SOF/RBV | 62.48 |
| SOF/DCV/RBV | 10.41 |
| SOF/DCV | 1.99 |

IQR, inter-quartile range; HCC, hepatocellular carcinoma; SOF, sofosbuvir; RBV, ribavirin; PEG-IFN, pegylated interferon; DCV, daclatasvir;

Supplementary table 2. Baseline characteristics of persons with and without incident HCC excluding 9 persons with hepatitis B virus coinfection.

| Variable | With HCC  N=40 | Without HCC  N=613 | P-value |
| --- | --- | --- | --- |
| Mean age, (SD) | 57.1 (8.4) | 48.6 (11.3) | <.0001 |
| Median age, IQR | 58 (52.5,64.5) | 50 (40,56) | <.0001 |
| Sex, % male | 45.00% | 49.10% | 0.61 |
| Weight, mean (SD) kg | 68.2 (12.3) | 72.1 (13) | 0.07 |
| Obesity (% obese) | 12.50% | 16.97% | 0.45 |
| Hypertension, % | 7.50% | 7.01% | 0.91 |
| Diabetes, % | 47.50% | 27.08% | 0.008 |
| Hepatitis B virus coinfection | 0.30% | 1.06% | 0.09 |
| Cirrhosis, % | 100.00% | 46.00% | <.0001 |
| Cirrhosis decompensated, % | 30.00% | 9.30% | 0.0004 |
| HCV RNA, median log_10_ IU/mL (IQR) | 6.2 (5.8,6.5) | 5.9 (5.2,6.5) | 0.44 |
| HCV genotype 3 | 95.00% | 90.54% | 0.31 |
| Sustained virologic response, % | 86.84% | 92.09% | 0.29 |
| Median time to HCC, weeks (IQR) | 27.5 (20.5,38.5) |  |  |
| Treatment regimen, % |  |  | 0.002 |
| SOF/RBV/PEG-IFN | 2.50% | 26.59% |  |
| SOF/RBV | 72.50% | 61.83% |  |
| SOF/DCV/RBV | 22.50% | 9.62% |  |
| SOF/DCV | 2.50% | 1.96% |  |

IQR, inter-quartile range; HCC, hepatocellular carcinoma; SOF, sofosbuvir; RBV, ribavirin; PEG-IFN, pegylated interferon; DCV, daclatasvir;

Supplementary table 3. Predictors of development of HCC (multivariable Cox regression model) excluding 9 persons with hepatitis B virus coinfection.

| Variable | HR (95% CI) | P-value |
| --- | --- | --- |
| Age, per 10 year increase | 1.71 (1.25,2.33) | 0.001 |
| Male sex | 1.15 (0.59,2.26) | 0.68 |
| Weight, each 5 kg increase | 0.85 (0.74,0.98) | 0.03 |
| Hypertension | 1.05 (0.31,3.58) | 0.94 |
| Diabetes | 1.48 (0.76,2.88) | 0.24 |
| HCV genotype 3 (comparator genotype non-3) | 1.36 (0.32,5.76) | 0.68 |
| Sustained virologic response | 0.35 (0.14,0.85) | 0.02 |
| Treatment regimen |  |  |
| SOF/RBV/PEG-IFN (comparator) |  |  |
| SOF/RBV | 6.66 (0.88,50.56) | 0.07 |
| SOF/DCV/RBV | 17.05 (2.09,139.47) | 0.01 |
| SOF/DCV | 8.65 (0.53,141.68) | 0.13 |

 SOF, sofosbuvir; RBV, ribavirin; PEG-IFN, pegylated interferon; DCV, daclatasvir;
